# Physicochemical Evolution, Biotransformation Behavior and Metabolomic Profiling of Hawthorn Pulp Co-Fermented by *Levilactobacillus brevis* and *Streptococcus thermophilus*

**DOI:** 10.3390/foods15132368

**Published:** 2026-07-03

**Authors:** Junhua Guo, Zengshuai Zhang, Shaoning Cheng, Xin Ma, Fen Wang, Tao Li

**Affiliations:** 1 School of Applied Engineering, Henan University of Science and Technology, Sanmenxia 472100, China; 2 College of Enology, Northwest A&F University, Yangling 712100, China; 3 College of Health, Yuncheng Vocational and Technical University, Yuncheng 044000, China

**Keywords:** hawthorn pulp, mixed fermentation, antioxidant activity, volatile compounds, metabolomics

## Abstract

Hawthorn (*Crataegus pinnatifida*) pulp is characterized by high levels of organic acids and polyphenols, yet its strong acidity limits sensory acceptance. In this study, *Streptococcus thermophilus* 540, *Lactobacillus brevis* 647, and their mixed culture (1:1) were applied to ferment hawthorn pulp. Changes in organic acids, individual phenolics, free amino acids, volatile compounds, antioxidant capacity, and global metabolic profiles were systematically investigated using targeted analysis combined with untargeted metabolomics. Fermentation markedly reshaped the metabolic composition of hawthorn pulp, with mixed fermentation exhibiting the most pronounced metabolic reprogramming. Co-fermentation significantly enhanced phenolic derivatives and aromatic amino acid-related metabolites, while promoting a more balanced distribution of organic acids and esters, contributing to improved antioxidant potential and flavor coordination. Multivariate analyses confirmed that mixed fermentation formed a distinct metabolic pattern rather than a simple additive effect of single strains, characterized by expanded metabolic diversity and pathway complementarity. These findings provide mechanistic insight into strain-dependent and synergistic metabolic modulation during fruit fermentation and support mixed lactic acid bacteria fermentation as a promising strategy for improving the functional and sensory quality of hawthorn-based products.

## 1. Introduction

Chinese hawthorn (*Crataegus pinnatifida* Bunge) is a representative fruit with dual attributes of food and medicine. Owing to its high contents of organic acids, polyphenols, flavonoids, and vitamin C, hawthorn has been well recognized for its nutritional and health-promoting properties, particularly in supporting digestive function, regulating lipid metabolism, and exerting antioxidant activity [1]. Fresh hawthorn (*Crataegus pinnatifida* Bunge) is characterized by high organic acid content and relatively low soluble sugar levels, resulting in an unbalanced sweetness–acidity profile that limits its direct palatability and consumer acceptance [2]. As a result, the processing and utilization of hawthorn have long faced a fundamental contradiction between its high functional potential and the insufficient sensory quality and stability of derived products [3]. These limitations have, to some extent, constrained the high-value application of hawthorn in fruit-based beverages and functional fermented foods. Consequently, developing mild and controllable processing strategies that can improve physicochemical properties while promoting the release and transformation of bioactive components has become a key focus in current research within food chemistry and functional food science.

Lactic acid bacteria fermentation has emerged as an important technological approach for the functional processing of fruits and vegetables, and has been increasingly applied in fruit pulps, juices, and plant-based beverage systems in recent years [4]. Compared with conventional thermal processing, LAB fermentation operates under mild conditions and can modify organic acid composition, promote the release of bioactive compounds, and enhance antioxidant properties through microbial metabolism [5,6]. However, most previous studies have focused on single-strain fermentations or endpoint comparisons, with limited attention given to dynamic metabolic changes during fermentation [7]. It is noteworthy that hawthorn is characterized by exceptionally high levels of organic acids and polyphenols, creating a unique matrix environment that exerts strong selective pressure on microbial metabolism [8]. On the one hand, the high initial acidity and complex phenolic structures may restrict the growth and metabolic potential of certain strains; on the other hand, these characteristics also provide opportunities for achieving synergistic fermentation through rational strain combinations [9]. Therefore, a systematic investigation of fermentation dynamics and metabolic responses is needed to better understand the functional modification of hawthorn pulp during LAB fermentation.

*Streptococcus thermophilus* is widely used in fermented food systems because of its rapid acid production and efficient utilization of fermentable sugars [10]. Its primary advantage lies in its ability to quickly reduce pH during the early stages of fermentation, thereby improving microbial safety and establishing a favorable sensory foundation of the substrate. In contrast, *Levilactobacillus brevis* exhibits stronger acid tolerance and greater metabolic versatility, with particular strengths in phenolic compound transformation, amino acid metabolism, and the release of flavor precursors [11]. However, studies on the co-fermentation of plant-based fruit matrices by *Levilactobacillus brevis* and *Streptococcus thermophilus* remain limited, and their combined effects on the physicochemical properties, biotransformation behavior, and overall metabolic profile of hawthorn pulp have not yet been clearly elucidated. As an advanced approach for deciphering metabolic changes in complex food systems, non-targeted metabolomics enables comprehensive characterization of metabolite composition and dynamic evolution, providing new insights into microbially driven biotransformation processes [12]. Nevertheless, in the context of lactic acid bacteria co-fermentation of hawthorn, studies that systematically integrate metabolomic data with physicochemical parameters, functional activities, and flavor attributes are still scarce.

Based on these considerations, this study selected hawthorn pulp as the model substrate and employed *Streptococcus thermophilus* 540 and *Levilactobacillus brevis* 647 as fermentation strains to establish single-strain and 1:1 co-fermentation system. The dynamic changes in total phenolics, total flavonoids, and in vitro antioxidant capacity during fermentation were systematically evaluated. In parallel, the compositional changes in organic acids, individual phenolic compounds, free amino acids, and volatile flavor compounds were analyzed to elucidate the regulatory effects of co-fermentation on bioactive and sensory-related constituents of hawthorn pulp. Furthermore, non-targeted metabolomics was applied to characterize the metabolic remodeling induced by co-fermentation and to identify key differential metabolites. This study aims to clarify the coordinated evolution of physicochemical properties, biotransformation behavior, and metabolic networks during the co-fermentation of hawthorn pulp by *L. brevis* and *S. thermophilus*. The findings are expected to provide a scientific basis for rational strain combination and precise quality control in fermented fruit-based products, while also offering new theoretical insights and technical strategies for the high-value utilization of hawthorn resources and the development of functional fermented foods.

## 2. Materials and Methods

### 2.1. Materials

*Lactobacillus brevis* 647 and *Streptococcus thermophilus* 540 were purchased from Guangzhou Microbial Culture Collection Center (Guangzhou, China). The MRS broth culture medium used in the experiment was purchased from Beijing Luqiao Technology Co., Ltd. (Beijing, China), and all met experimental standards.

DNS reagent, ABTS, DPPH reagent, Folin’s reagent, sodium nitrite, glucose and other chemical reagents were all analytical grade and purchased from Tianjin Kemeio Chemical Reagent Co., Ltd. (Tianjin, China); methanol, acetic acid, phosphate, acetonitrile and other reagents used for chromatographic detection were chromatographic grade and purchased from Merck AG, Darmstadt, Germany. Organic acid standards such as malic acid, citric acid, tartaric acid, lactic acid, and oxalic acid were all obtained from Tanmo Quality Inspection-Standard Material Center (Changzhou, China).

### 2.2. Preparation of Hawthorn Fruit Pulp by Microbial Fermentation

Fresh hawthorn fruits (Crataegus pinnatifida Bunge) were sourced from Chengde, Hebei Province, China. Fruits without obvious defects were selected based on ripeness and thoroughly washed with distilled water. The pits were removed by hand, and the fruits were steamed at 100 °C for 20 min. After cooling, they were homogenized in a sterile high-speed mixer to obtain hawthorn pulp. The pulp was filtered through double-layer sterile gauze to remove coarse particles. The initial pH and Soluble solid content (SSC) content were measured to be pH 3.05 and 8.5 °Brix, respectively. The pH and SSC of the hawthorn pulp were adjusted to (4.0 ± 0.1) and 13.0 ± 0.5 °Brix, respectively, by adding sodium bicarbonate and white sugar. The pulp was then pasteurized in a water bath at 85 °C for 15 min and cooled to room temperature before use. Activated *Streptococcus thermophilus* and *Lactobacillus brevis* were inoculated into hawthorn pulp, and the viable bacterial count was cultured to 10^9^ CFU/mL before being used as seed culture medium.

At an inoculation rate of 3% (*v*/*v*), *Lactobacillus brevis* seed culture, *Streptococcus thermophilus* seed culture, and a 1:1 mixed culture was inoculated into hawthorn pulp and fermented at 37 °C for 36 h. Fermentation was carried out in a 5.0 L sterile fermenter. The system was operated under anaerobic conditions. The fermenter was placed in a thermostatic incubator and maintained at 37 °C throughout the fermentation process. Samples were taken from all experimental groups every 6 h and immediately stored at −80 °C for later analysis. Hawthorn pulp without inoculation was used as a blank control under the same conditions to eliminate the influence of the substrate itself.

### 2.3. Determination of Physicochemical Indicators

#### 2.3.1. Determination of pH, Total Acid, and Viable Count

After fermentation, 1.0 mL of fermentation broth was serially diluted in sterile physiological saline solution (0.85%, *w*/*v*) using a ten-fold serial dilution method. The total viable cell count was determined using the dilution plate method. Before determining other physicochemical indicators, the fermentation samples were centrifuged at 6000× *g* for 10 min at 4 °C to remove insoluble particles. The resulting supernatant was collected and used for subsequent physicochemical analyses. The pH value and soluble solids content were measured using a pH meter and an automatic polarimeter, respectively. The total acid content was expressed as lactic acid content.

#### 2.3.2. Determination of Reducing Sugars

Take 1.0 mL of sample solution, add 1.0 mL of DNS reagent and mix thoroughly. Heat in a boiling water bath for 5 min, then immediately cool in cold water to room temperature. Add distilled water to a final volume of 10 mL and shake well. Zero the volume with a blank tube and measure the absorbance at 540 nm using a spectrophotometer (Cary 7000, Agilent, Santa Clara, CA, USA) with 1 cm quartz cuvettes.

#### 2.3.3. Determination of Total Phenolics

Total phenolic content (TPC) was determined using the Folin–Ciocalteu method. Gallic acid was used as the calibration standard, and the results were expressed as mg GAE/L. Briefly, 0.2 mL of sample solution was mixed with 0.5 mL of Folin–Ciocalteu reagent, followed by 0.5 mL of 5% sodium carbonate solution and 0.8 mL of deionized water. The mixture was thoroughly mixed after each addition and incubated at room temperature for 1 h. Absorbance was measured at 760 nm using a spectrophotometer with 1 cm quartz cuvettes.

#### 2.3.4. Determination of Total Flavonoids

Total flavonoid content (TFC) was determined using the sodium nitrite–aluminum nitrate colorimetric method. Rutin was used as the calibration standard, and the results were expressed as mg RE/L. Briefly, 0.2 mL of sample solution was mixed with 0.3 mL of 5% sodium nitrite solution and allowed to stand for 5 min. Subsequently, 0.3 mL of 10% aluminum nitrate solution was added and reacted for 6 min. Finally, 2.0 mL of 1 mol/L sodium hydroxide solution was added. After incubation for 15 min, absorbance was measured at 510 nm using a spectrophotometer with 1 cm quartz cuvettes.

### 2.4. Determination of Organic Acids and Vitamin C

Organic acids and vitamin C were analyzed using an HPLC system (Shimadzu LC-20A, Kyoto, Japan). Separation was performed on an InertSustain AQ-C18 column (250 mm × 4.6 mm, 5 μm) at 30 °C. The mobile phase consisted of 0.02 mol/L diammonium hydrogen phosphate solution (pH 2.40), delivered at a flow rate of 1.0 mL/min under isocratic conditions. The total run time was 20 min. The detection wavelength was set at 210 nm. Calibration curves were constructed using at least 3 concentration levels for each standard compound over appropriate ranges. All calibration curves showed good linearity with correlation coefficients (R^2^) ≥ 0.99.

### 2.5. Determination of Amino Acids

Amino acid analysis was performed using acid hydrolysis followed by ion-exchange chromatography. Briefly, 2.0 mL of fermentation sample was transferred into a hydrolysis tube, and 6 mol/L hydrochloric acid was added to a final volume of 10.0 mL. Three to four drops of phenol were added as an antioxidant. The tubes were flushed with nitrogen, sealed, and hydrolyzed at 110 °C for 22 h. After hydrolysis, the samples were cooled to room temperature, diluted to volume, filtered, and evaporated to dryness. The residues were reconstituted with sodium citrate buffer for analysis. A fully automatic amino acid analyzer equipped with an ion-exchange column was used for separation. Post-column derivatization with ninhydrin was performed, and detection was carried out at 570 nm and 440 nm. Quantification was conducted using external amino acid standards. It should be noted that acid hydrolysis may lead to degradation, oxidation, or transformation of certain amino acids.

### 2.6. Determination of Antioxidant Activity

#### 2.6.1. Determination of DPPH Radical Scavenging Ability

The DPPH radical solution was freshly prepared by dissolving DPPH in methanol to a final concentration of 0.2 mmol/L and stored in the dark before use. Then, 2.0 mL of diluted sample was mixed with 2.0 mL of DPPH solution, incubated in the dark for 30 min, and measured at 517 nm using a UV–Vis spectrophotometer.

#### 2.6.2. Determination of ABTS+ Radical Scavenging Ability

ABTS radical stock solution was prepared: an equal volume of 7.4 mmol/L ABTS solution was mixed thoroughly with 2.6 mmol/L K_2_S_2_O_8_ solution and allowed to stand at room temperature in the dark for 12 h to allow sufficient free radical generation. The stock solution was then diluted with 95% ethanol until the absorbance at 734 nm reached 0.70 ± 0.02, yielding a 7 mmol/L ABTS•+ working solution. An amount of 0.1 mL of the diluted hawthorn juice sample was added to 3.9 mL of the ABTS•+ working solution. After thorough mixing, the solution was incubated at room temperature in the dark for 10 min. After incubation, the absorbance was measured at 734 nm, and the scavenging capacity of the sample for ABTS+ free radicals was calculated.

#### 2.6.3. Determination of Hydroxyl Radical Scavenging Rate

An amount of 1.0 mL ethanol-salicylic acid solution, 1 mL ferrous sulfate solution, 1 mL hydrogen peroxide solution, and finally, 1.0 mL of the sample solution were added together. After thorough mixing, the solution was incubated at 37 °C for 30 min to allow hydroxyl radicals to fully generate and react with the antioxidant components in the sample. After the reaction was completed, the absorbance was measured at a wavelength of 510 nm, and the scavenging ability of the sample for hydroxyl radicals was calculated based on the absorbance.

### 2.7. Determination of Monomeric Phenols

We took 20.0 mL of juice sample diluted 10 times and extracted it three times with 60 mL of ethyl acetate. We collected the organic phase after each extraction and combined all extracted organic phases. We concentrated the combined organic phase under reduced pressure at 37 °C until completely dry. The sample was dissolved in 2.0 mL of methanol and filtered through a 0.22 μm PES membrane filter prior to HPLC analysis. An aliquot of 10.0 μL was injected into the HPLC system for quantification.

The mobile phase consisted of 0.02% formic acid (Phase A) and methanol (Phase B). Gradient settings were as follows: 0–5 min, 10% Phase B; 5–10 min, 20% Phase B; 10–20 min, 35% Phase B; 20–35 min, 40% Phase B; 35–40 min, 75% Phase B; 40–45 min, 10% Phase B. The flow rate was 1 mL/min, the column oven temperature was 30 °C, and the detection wavelength was 280 nm.

### 2.8. Metabolomics Determination

We precisely transferred 1.0 mL of fermentation broth into a centrifuge tube, and added 4.0 mL of pre-cooled 80% methanol aqueous solution to precipitate proteins. The mixture was vortexed thoroughly and centrifuged at 12,000× *g* for 15 min at 4 °C. The supernatant was filtered through a 0.22 μm organic membrane filter. For quality control (QC), equal aliquots of all samples were pooled to generate a QC sample, which was used to monitor instrument stability and data reproducibility. QC samples were injected at regular intervals (one QC injection after every 10 analytical samples) throughout the analytical sequence.

Metabolomic profiling was performed using an UHPLC-Q Exactive Orbitrap mass spectrometer (Thermo Fisher Scientific, Waltham, MA, USA). Chromatographic separation was achieved on a C18 column (2.1 × 100 mm, 1.7 μm) at 40 °C with an injection volume of 5 μL. The mobile phases consisted of 0.1% formic acid in water (A) and acetonitrile (B), with a flow rate of 0.3 mL/min. The gradient program was set as follows: 0–2 min, 5% B; 2–15 min, 5–95% B; 15–18 min, 95% B; 18–20 min, 95–5% B; 20–25 min, 5% B.

The mass spectrometry was operated in both positive and negative electrospray ionization modes (ESI+ and ESI−). Full scan data were acquired over a mass range of *m*/*z* 70–1050 at a resolution of 70,000 (*m*/*z* 200). The automatic gain control (AGC) target was set to 3 × 10^6^, with a maximum injection time of 100 ms. Data-dependent MS/MS acquisition was performed using stepped normalized collision energy (NCE 20, 40, and 60 eV). Metabolite annotation was performed by matching accurate mass (mass error ≤ 5 ppm), MS/MS fragmentation patterns, and retention time against public databases KEGG.

Multivariate statistical analyses were performed using SIMCA software (version 14.01). Principal component analysis (PCA) and partial least squares-discriminant analysis (PLS-DA) were conducted. Model robustness was evaluated using R^2^X, R^2^Y, and Q^2^ parameters, and validated by 200-permutation testing. Features were considered statistically significant at variable importance in projection (VIP) > 1 and *p* < 0.05. To control false discovery rate, Benjamini–Hochberg correction was applied, and metabolites with adjusted *p*-values (FDR) < 0.05 were considered significantly different.

### 2.9. Aroma Component Determination

An amount of 5.0 mL of hawthorn fruit pulp sample was placed in a 20.0 mL headspace vial, and an appropriate amount of sodium chloride solid was added to promote the release of volatile aroma components in the sample. The sealed sample was placed in a constant temperature environment of 40 °C for 15 min to allow the aroma components to reach equilibrium in the gas-liquid two-phase system before analysis.

Aroma component analysis was performed using gas chromatography-mass spectrometry (GC-MS, Agilent 7890 GC coupled with 5977 MSD, Agilent Technologies, USA). Gas chromatography conditions were: DB-Wax capillary column (30 m × 0.25 mm × 0.25 μm), inlet temperature 250 °C, splitless injection. The temperature rising program was as follows: hold at 40 °C for 3 min, increase to 200 °C at a rate of 5 °C/min, and then increase to 240 °C at a rate of 10 °C/min and maintain for 5 min. Quantification of aroma components was performed using the peak area normalization method for semi-quantitative analysis, with 2-octanol as the internal standard, and the final results are expressed in μg/L.

### 2.10. Statistical Analysis

All experimental data are expressed as mean ± standard deviation (Mean ± SD, n = 3). SPSS 24.0 software (SPSS Inc., Chicago, IL, USA) was used for statistical analysis. One-way analysis of variance (ANOVA) and Duncan’s multiple range test were used for comparisons between multiple groups, and independent samples *t* tests were used for comparisons between two groups. Graphs were drawn using GraphPad Prism 6.0 (GraphPad Software, San Diego, CA, USA) and Origin 2022 (OriginLab Corporation, Northampton, MA, USA) software.

## 3. Results and Discussion

### 3.1. Effects of Different Fermentation Strategies on the Basic Physicochemical Properties of Hawthorn Pulp

The 36 h fermentation period was selected to encompass the complete fermentation process under the tested conditions. Most measured parameters exhibited rapid changes during the early and middle stages of fermentation, whereas their variation became less pronounced after 30 h, suggesting progressive stabilization of the fermentation system. Therefore, the final sampling point at 36 h was intended to characterize the late fermentation stage rather than to represent an optimized fermentation endpoint.

The results presented in Table 1 illustrate pronounced changes in the basic physicochemical properties of hawthorn pulp during fermentation. The initial pH of hawthorn pulp was approximately 3.96, which is characteristic of a highly acidic fruit-based matrix. As fermentation proceeded, the pH values of all treatments decreased over time, accompanied by a continuous increase in titratable acidity, indicating that lactic acid bacteria were able to rapidly adapt and initiate metabolic activity even under low initial pH conditions [13]. *S. thermophilus* exhibited a relatively fast acidification capacity during the early stage of fermentation. Its pH decreased to 3.72 within the first 6 h, reflecting its strong ability to rapidly produce organic acids. In contrast, *L. brevis*, owing to its higher acid tolerance, showed a more gradual but sustained decline in pH throughout the entire fermentation process [14]. Notably, the pH evolution in the co-fermentation system closely resembled that of *S. thermophilus* during the first 12 h, while gradually converging toward the trend observed for *L. brevis* at the middle and later stages of fermentation. This observation suggests the presence of stage-dependent dominance of the two strains during co-fermentation.

Corresponding to the changes in pH, titratable acidity increased markedly in all fermentation groups. Among the single-strain fermentations, the *L. brevis* group consistently exhibited higher total acid levels than the *S. thermophilus* group throughout the fermentation period, with final titratable acidity reaching 9.56–10.27 g/L. This result indicates a stronger capacity of *L. brevis* for organic acid accumulation [15]. At the end of fermentation, the total acidity of the co-fermentation system fell between those of the two single-strain fermentations, suggesting that the mixed culture did not compromise organic acid production. Instead, it may have achieved a more stable acidification process through metabolic complementarity. An appropriate increase in acidity is beneficial for enhancing flavor complexity and product stability, whereas excessive acidification can negatively affect sensory acceptance [16]. In this regard, co-fermentation exhibited a comparatively moderate and controllable acid production profile.

Changes in soluble solids and reducing sugar contents further reflected differences in carbon source utilization among the fermentation modes. The decrease in soluble solids and reducing sugars indicated active carbohydrate utilization by lactic acid bacteria, reflecting their role in fermentative carbohydrate metabolism. Notably, the co-fermentation system displayed the greatest extent of reducing sugar consumption, with reducing sugar contents decreasing to 8.54–8.37 mg/mL after 30 h, which was clearly lower than those observed in the single-strain fermentations. This observation may reflect differential carbon utilization patterns in the mixed-culture system; however, further analysis of individual sugar components would be required to substantiate this hypothesis [17].

All three fermentation modes maintained high viable cell counts throughout fermentation, with populations remaining above 10^8^–10^9^ CFU/mL. These results indicate that both *S. thermophilus* and *L. brevis* were able to grow and remain metabolically active in the hawthorn pulp substrate. The mixed-culture fermentation group also maintained consistently high viable counts during fermentation, suggesting that the co-fermentation system provided a favorable environment for microbial survival. However, because only total viable counts were determined, the relative abundance and population dynamics of the individual strains could not be assessed. Changes in total phenols and total flavonoids showed a clear fermentation method dependence. Total phenolic content remained relatively stable throughout fermentation, suggesting that lactic acid fermentation did not significantly affect overall phenolic levels under the present conditions [18]. In contrast, total flavonoid content exhibited more pronounced fluctuations during fermentation. A rapid increase was observed in the L. brevis fermentation group within the first 6 h, whereas the increase in the S. thermophilus group was comparatively limited. The mixed-culture fermentation also showed an early increase in total flavonoids and maintained relatively high levels during the later stages of fermentation. The observed fluctuations may reflect the dynamic balance between the release and transformation of flavonoid compounds during fermentation. Previous studies have suggested that microbial enzymatic activities can enhance the extractability of matrix-bound flavonoids in the early stages of fermentation, while subsequent biotransformation or degradation of certain flavonoid structures may alter the overall flavonoid profile [19]. Since only total flavonoid content was determined in the present study, the specific mechanisms underlying these changes require further investigation through targeted flavonoid analysis.

### 3.2. Effects of Different Fermentation Modes on Antioxidant Activity and Vitamin C Content of Hawthorn Pulp

**Figure 1:**
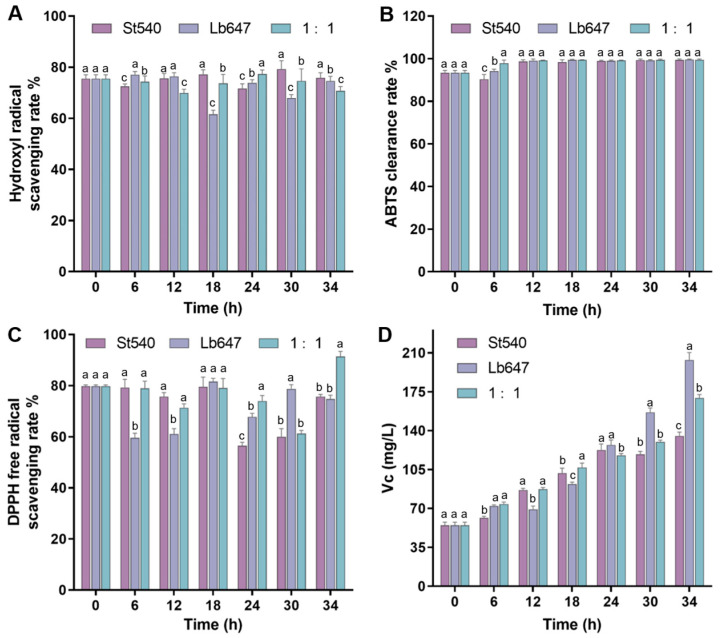
Effects of different fermentation modes on antioxidant activity and vitamin C content of hawthorn pulp. (**A**) hydroxyl radical scavenging rate; (**B**) ABTS radical scavenging rate; (**C**) DPPH radical scavenging rate; (**D**) Vitamin C content. Different lowercase letters in the figure represent significant differences (*p* < 0.05).

As shown in Figure 1, hawthorn pulp exhibited a high baseline antioxidant capacity, with hydroxyl radical, ABTS, and DPPH scavenging activities of 75.56%, 93.44%, and 79.81%, respectively, and a vitamin C content of 54.89 mg/L. After 6 h of fermentation with *S. thermophilus*, hydroxyl radical and ABTS scavenging activities slightly decreased to 72.52% and 90.37%, respectively, while DPPH scavenging activity remained nearly unchanged at 79.29%. In contrast, vitamin C (Vc) content increased significantly to 61.59 mg/L. Fermentation with *L. brevis* for 6 h resulted in an increase in hydroxyl radical scavenging activity to 77.12% and a stable ABTS scavenging activity of 94.24%, accompanied by a marked decrease in DPPH scavenging activity to 59.62%, indicating selective modulation of antioxidant responses. Notably, co-fermentation markedly enhanced antioxidant performance at the early stage, with ABTS scavenging activity increasing to 97.89% and vitamin C content reaching 73.91 mg/L, both exceeding those of the single-strain fermentations. These findings suggest that co-fermentation promotes the rapid accumulation of antioxidant-active compounds in hawthorn pulp.

At 18 h of fermentation, the accumulation of vitamin C became more pronounced, reaching 106.87 mg/L in the co-fermentation system, which was higher than that observed for *S. thermophilus* (101.99 mg/L) and *L. brevis* (92.06 mg/L). During this stage, ABTS scavenging activity remained consistently high across all fermentation groups, indicating the establishment of a relatively stable antioxidant system in the mid-fermentation phase [20]. DPPH scavenging activity peaked at 81.64% in the *L. brevis* group, suggesting a transient re-accumulation of DPPH-sensitive antioxidant components. At 24 h, DPPH scavenging activity in the *S. thermophilus* group decreased markedly to 56.52%, whereas co-fermentation maintained a higher level at 74.02%, highlighting the advantage of mixed cultures in sustaining multiple antioxidant activities. Throughout the later fermentation stage, ABTS scavenging activity remained above 99%, indicating a saturated response to fermentation-induced antioxidant enhancement [21]. In contrast, hydroxyl radical scavenging activity showed greater fluctuations, with values at 30 h reaching 79.31% for *S. thermophilus* and 67.95% and 74.61% for *L. brevis* and co-fermentation, respectively, suggesting a stronger dependence on the instantaneous composition of active antioxidant compounds [22].

At 30 h of fermentation, the vitamin C content in the *L. brevis* group increased to 156.41 mg/L, which was higher than that observed in the S. thermophilus group (118.89 mg/L) and the co-fermentation group (129.84 mg/L). At 34 h, vitamin C reached 203.60 mg/L in the *L. brevis* group, compared with 169.38 mg/L and 135.33 mg/L in the co-fermentation and *S. thermophilus* groups, respectively. The elevated vitamin C levels observed during fermentation may be associated with reduced oxidative degradation under acidic conditions and enhanced preservation of ascorbic acid. However, because only ascorbic acid was quantified and no analysis of dehydroascorbic acid or matrix interference was performed, the underlying mechanisms responsible for the apparent increase require further investigation [23].

### 3.3. Effects of Different Fermentation Modes on Organic Acid Profiles of Hawthorn Pulp

As shown in Table 2, tartaric acid (814.70 mg/L), malic acid (514.13 mg/L), and citric acid (356.59 mg/L) were the predominant organic acids detected in hawthorn pulp. Lactic acid was not detected in the unfermented sample, indicating that the acidity of the raw hawthorn pulp was primarily attributable to naturally occurring fruit organic acids. After 6 h of fermentation, malic acid in the *S. thermophilus* 540 and co-fermentation groups decreased to below the detection limit, whereas 42.34 mg/L remained in the *L. brevis* 647 group, suggesting a greater reduction in malic acid during the early fermentation stage in the former treatments. With prolonged fermentation, malic acid gradually reappeared in all treatments, reaching 273.35–277.81 mg/L at 18 h and further increasing to 311.85 mg/L in the co-fermentation system at 30 h. The observed recovery of malic acid indicates that its concentration was dynamically regulated during fermentation, although the underlying mechanisms could not be determined from the present study.

Citric acid content in the *S. thermophilus* fermentation decreased rapidly from 356.59 mg/L to 100.16 mg/L within 6 h and remained at a low level of 82.88–90.67 mg/L thereafter, indicating a sustained capacity for citrate utilization [24]. In contrast, *L. brevis* showed pronounced citrate accumulation at 12 and 18 h, reaching 386.38 and 456.21 mg/L, respectively, which may be associated with the release of bound citrate or modulation of citrate-related metabolic pathways. In the co-fermentation system, citric acid was maintained at a relatively stable level of approximately 90-100 mg/L, suggesting a balance between citrate consumption and release that contributed to a more stable acid profile [25].

Tartaric acid in the *S. thermophilus* group gradually decreased from 814.70 mg/L to 656.42 mg/L at 30 h, whereas the *L. brevis* group-maintained levels ranging from 669.73 to 844.02 mg/L during the middle and late stages of fermentation. Notably, co-fermentation led to an early increase in tartaric acid to 926.33 mg/L at 6 h, and relatively high levels were still observed at 30 h and 34 h, reaching 777.96 and 801.46 mg/L, respectively. The higher and more stable tartaric acid content in the co-fermentation system likely contributed to preserving the characteristic mild fruit acidity of hawthorn pulp and preventing an overly simplified acid profile dominated by lactic acid [26].

Lactic acid was the organic acid showing the most pronounced changes during fermentation. *S. thermophilus* produced 341.72 mg/L lactic acid within 6 h, whereas higher levels were observed in the *L. brevis* and co-fermentation groups, reaching 515.35 and 564.42 mg/L, respectively. As fermentation progressed, lactic acid continued to accumulate, with the co-fermentation system reaching 1325.49 mg/L at 24 h, notably higher than that of *S. thermophilus* (1164.24 mg/L). By 34 h, lactic acid content in the co-fermentation further increased to 1934.98 mg/L, slightly exceeding that in the *S. thermophilus* (1917.74 mg/L) and *L. brevis* (1799.43 mg/L) groups. These results indicate that the mixed culture maintained a high and stable lactic acid production capacity throughout fermentation, reflecting enhanced co-fermentation effects between the two strains in carbon utilization and energy metabolism [27].

Regarding minor organic acids, oxalic acid increased markedly during the early stage of fermentation, reaching 75.80 mg/L and 75.55 mg/L at 6 h in the *S. thermophilus* and *L. brevis* groups, respectively. In contrast, oxalic acid in the co-fermentation system gradually decreased thereafter, dropping to 22.11 mg/L and 28.32 mg/L at 24 h and 30 h, respectively, which were lower than those in the single-strain fermentations. This suggests a potential buffering or regulatory effect of co-fermentation on oxalic acid accumulation. Shikimic acid exhibited an overall increasing trend during fermentation, with co-fermentation reaching 44.36 mg/L at 30 h and 48.76 mg/L at 34 h, comparable to or slightly lower than the single-strain groups, indicating sustained activation of aromatic metabolism-related pathways. Quinic acid showed relatively minor fluctuations across fermentation modes, remaining stable at 26–31 mg/L in the co-fermentation system. Given that quinic acid is a precursor involved in phenolic biosynthetic pathways, its stability may reflect a relatively stable metabolic background during fermentation; however, the direct relationship with polyphenol metabolism requires further verification.

### 3.4. Effects of Different Fermentation Modes on the Formation of Individual Phenolic Compounds in Hawthorn Pulp

Based on the results presented in Table 3, the most pronounced changes in individual phenolic compounds occurred during the early stage of fermentation (0–6 h), particularly for chlorogenic acid, caffeic acid, and flavanol-related compounds. In the unfermented hawthorn pulp, chlorogenic acid was present at 18.67 mg/L. After 6 h of fermentation with *S. thermophilus*, *L. brevis*, and the mixed culture, its content increased significantly to 47.67, 47.14, and 51.37 mg/L, respectively, representing more than a 2.5-fold increase compared with the initial level. These results indicate that lactic acid fermentation at the early stage effectively promoted the release of bound chlorogenic acid, which may be associated with cell wall degradation or the activation of ester bond-hydrolyzing enzyme systems [28]. Notably, the mixed fermentation exhibited the highest chlorogenic acid content at this stage, suggesting an enhanced co-fermentation effect of the two strains in enhancing phenolic acid release. In contrast, caffeic acid showed a marked decrease during the early fermentation phase. Its initial content of 58.41 mg/L dropped sharply after 6 h to 6.88 mg/L and 11.73 mg/L in the *S. thermophilus* and *L. brevis* fermentations, respectively, while the mixed fermentation resulted in a level of 10.46 mg/L. In all cases, the reduction exceeded 80%, indicating rapid transformation or utilization of caffeic acid during the early stage of lactic acid fermentation.

During the mid-fermentation stage (12–18 h), chlorogenic acid in the *S. thermophilus* group decreased markedly to 19.78 mg/L, whereas higher levels were maintained in the *L. brevis* and co-fermentation groups at 49.19 and 47.31 mg/L, respectively, indicating a relative advantage of *L. brevis* in preserving or regenerating chlorogenic acid [29]. At the same time, caffeic acid rebounded at 12 h in the *S. thermophilus* and *L. brevis* fermentations to 62.87 and 56.29 mg/L, respectively, while remaining low in the co-fermentation system at 7.85 mg/L. At 30 h, chlorogenic acid converged to a narrow range of 17.07–17.59 mg/L in all groups, close to the initial level, indicating a dynamic balance between release and transformation. Catechin and epicatechin showed a slight recovery at the late stage, with epicatechin in the co-fermentation system reaching 65.59 and 58.27 mg/L at 30 and 34 h, respectively, values that were comparable to or higher than those in the single-strain fermentations. This trend suggests that co-fermentation exerted a protective or sustained-release effect on flavanol compounds.

Rutin exhibited minimal variation throughout fermentation, remaining stable at 14–16 mg/L, indicating high structural stability and limited direct utilization by lactic acid bacteria. Kaempferol decreased markedly during the early fermentation stage and subsequently recovered to 163–176 mg/L in the later stage, suggesting an initial transformation followed by metabolic re-equilibration. Ferulic acid remained relatively constant at approximately 12–14 mg/L during late fermentation, with consistently higher levels observed in the co-fermentation system, implying that mixed cultures confer an advantage in maintaining the stability of low-molecular-weight phenolic acids [30].

### 3.5. Effects of Different Fermentation Modes on Free Amino Acids in Hawthorn Pulp

As shown in Table 4, the composition and dynamic changes of free amino acids in hawthorn pulp were markedly influenced by fermentation. In the unfermented pulp, most free amino acids were present at low levels, generally ranging from 0.2 to 3.3 mg/L. Glutamic acid at 3.321 mg/L, aspartic acid at 2.656 mg/L, leucine at 1.694 mg/L, and lysine at 1.706 mg/L were relatively abundant. These results indicate that hawthorn pulp is not inherently rich in free amino acids, and that nitrogen is mainly present in the form of bound proteins or peptides [2]. After 6 h of fermentation, glutamic acid levels in the *S. thermophilus*, *L. brevis*, and mixed-culture fermentations were 3.365, 3.364, and 3.364 mg/L, respectively, showing little change compared with the initial values. Similarly, amino acids such as aspartic acid, serine, and threonine remained close to their original concentrations. This suggests that, during the early stage of fermentation, lactic acid bacteria primarily relied on soluble carbohydrates as energy sources, while amino acid metabolism had not yet become dominant [31]. At the same time, it reflects the relatively low hydrolysis rate of protein substrates in hawthorn pulp.

At 12 h of fermentation, alanine increased to 1.513 mg/L and 1.510 mg/L in the *S. thermophilus* and *L. brevis* fermentations, respectively, whereas a lower level of 1.156 mg/L was observed in the mixed culture, indicating a slightly stronger alanine release in the single-strain systems at this stage. Notably, taste-active amino acids exhibited distinct regulatory patterns during fermentation [32]. Glutamic acid remained largely stable throughout the process, fluctuating within a narrow range of 3.32 to 3.36 mg/L. Only a transient decrease was observed in the *S. thermophilus* group at 24 h to 3.324 mg/L, followed by recovery to 3.341 mg/L at 30 h, highlighting the metabolic stability of glutamic acid during lactic acid bacterial fermentation. Sweet- or mild-tasting amino acids, such as glycine and alanine, showed a slight increase during the middle and late stages of fermentation. This accumulation may help to offset the sharp acidity caused by lactic acid production, thereby improving overall flavor balance. In the late fermentation stage, leucine, tyrosine, and phenylalanine in the *S. thermophilus* group increased to 1.749, 0.799, and 1.101 mg/L, respectively, all exceeding their levels at 18 h. In the *L. brevis* fermentation, phenylalanine and tyrosine reached 1.090 and 0.821 mg/L, respectively. In contrast, the mixed fermentation maintained relatively stable levels for most amino acids. For example, at 30 h, leucine and tyrosine were 1.743 and 0.818 mg/L, respectively, suggesting that amino acid formation and consumption were more balanced in the co-fermentation system.

### 3.6. Effects of Different Fermentation Modes on the Volatile Components of Hawthorn Pulp

**Figure 2:**
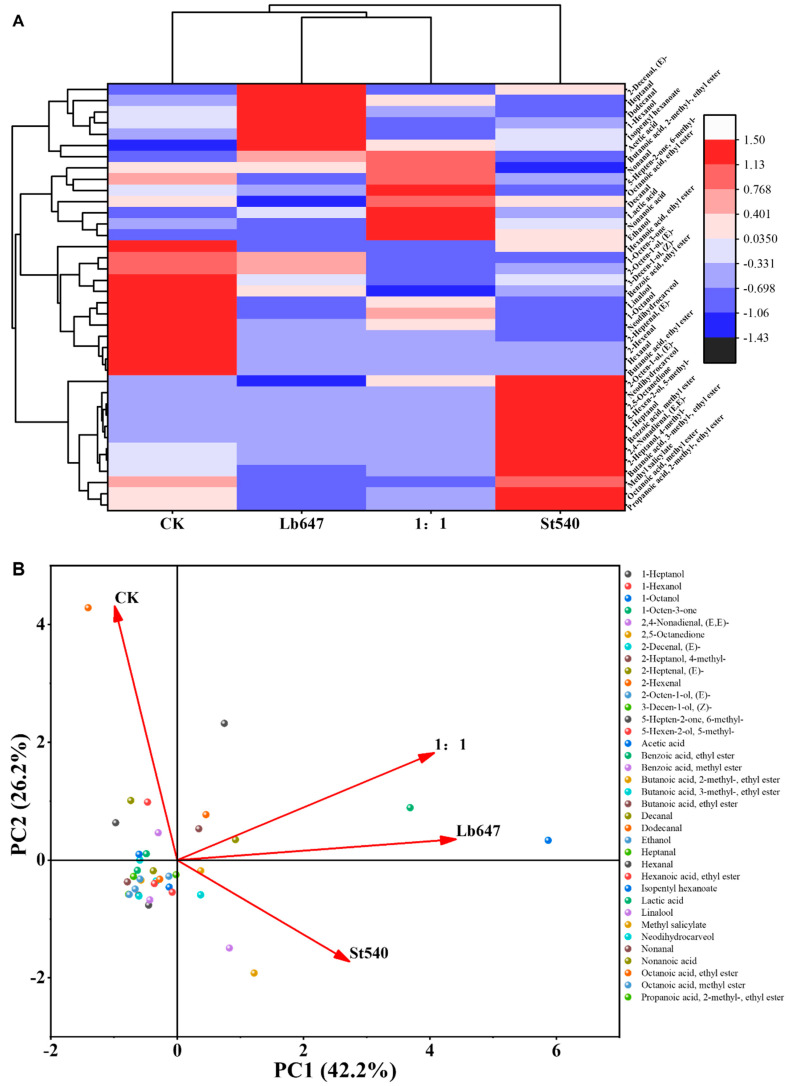
Effect of probiotic fermentation on the volatile components of hawthorn pulp. (**A**) Cluster heatmap analysis. (**B**) PCA analysis.

Figure 2A,B show that a total of 48 volatile compounds were identified in hawthorn pulp, including esters, organic acids, and other volatile components. Different fermentation methods significantly altered the total amount and composition of volatile substances in hawthorn pulp, exhibiting clear strain dependence and enhanced co-fermentation effects. The total volatile content in the unfermented control group (CK) was 49.80 μg/L, while those in *Streptococcus thermophilus* 540 (St540), *Lactobacillus brevis* 647 (Lb647), and mixed fermentation reached 312.00 μg/L, 124.97 μg/L, and 231.14 μg/L, respectively. Among the identified compounds, esters were significantly enriched after fermentation, whereas no lactate-derived esters were detected under the present experimental conditions. This may be attributed to strain-specific metabolic pathways or limited precursor availability for esterification reactions in the hawthorn matrix. Overall, all fermentation treatments showed higher volatile compound levels than the control group, indicating that lactic acid bacteria fermentation enhances volatile metabolite formation in hawthorn pulp.

Esters and organic acids represented the two volatile groups most strongly influenced by fermentation. Esters are widely recognized as key contributors to fruity and pleasant aroma characteristics in fermented foods and beverages. Compared with the control group, ester levels increased markedly after fermentation, particularly in the St540 treatment, where the total ester content reached 62.39 μg/L, approximately 6.3-fold higher than that of the control. This increase suggests that fermentation promoted the formation of aroma-active compounds associated with improved flavor quality. The enhanced ester production may be related to microbial metabolism of carbohydrates and organic acids, which generates volatile precursors that participate in ester biosynthesis. Previous studies have shown that lactic acid bacteria can influence ester accumulation by altering substrate availability and metabolic fluxes during fermentation. However, because esterification-related enzymes and precursor metabolites were not analyzed in the present study, the specific mechanisms responsible for ester formation remain to be clarified.

Organic acids also increased substantially following fermentation. In addition to contributing directly to sourness perception, organic acids play an important role in shaping overall flavor balance and may indirectly influence aroma expression by modifying the physicochemical environment of the fermentation system [33]. The St540 treatment exhibited the highest organic acid content, suggesting a stronger capacity for acid metabolism under the tested conditions. Although the mixed-culture fermentation produced lower total ester levels than St540 monoculture fermentation, it generated considerably higher ester concentrations than the Lb647 treatment and displayed a more balanced distribution of volatile compounds. This observation suggests that co-fermentation altered the volatile profile in a manner that may contribute to greater aroma complexity. Nevertheless, sensory evaluation and targeted aroma activity analyses would be required to confirm the actual sensory impact of these compositional changes.

A limitation of the present study is that flavor-related conclusions were primarily based on changes in volatile compounds and metabolite profiles, whereas no sensory evaluation or consumer acceptance assessment was performed. Although the observed changes in esters, organic acids, and other aroma-related metabolites suggest potential modifications in flavor characteristics, the actual sensory consequences of these compositional changes remain uncertain. Future studies combining sensory evaluation, odor activity value analysis, GC–olfactometry, and consumer testing are needed to establish direct links between chemical composition and perceived flavor quality.

### 3.7. Non-Targeted Metabolomic Analysis of Metabolite Composition and Metabolic Patterns in Fermented Hawthorn Pulp

**Figure 3:**
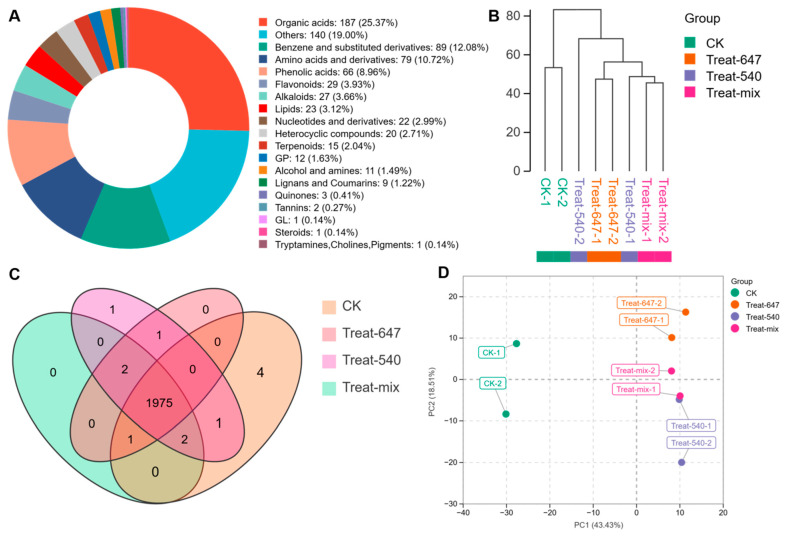

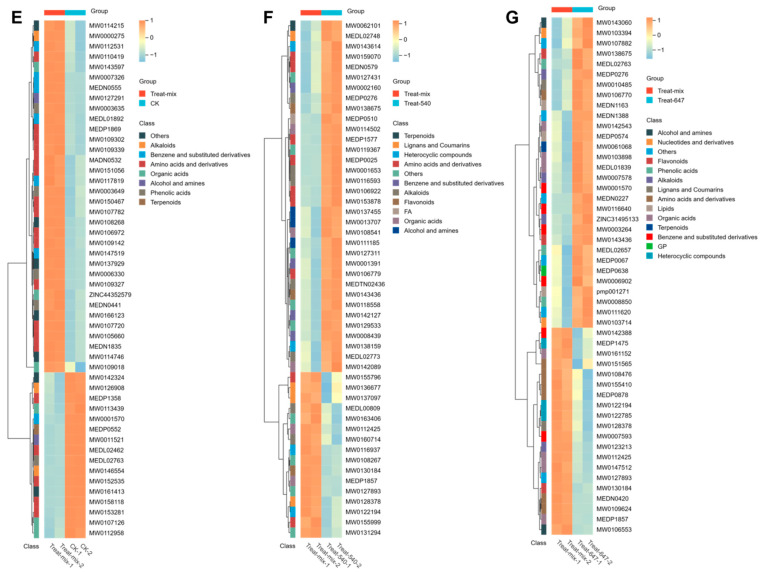
Non-targeted metabolomic analysis of metabolite composition and metabolic patterns in fermented hawthorn pulp. (**A**) Types of metabolites; (**B**) Sample hierarchical clustering tree. (**C**) Venn diagram analysis; (**D**) PCA analysis of differential metabolites; Clustering heatmap analysis of differential metabolites among groups with different fermentation methods (**E**) Mix vs. CK, (**F**) Mix vs. St540, (**G**) Mix vs. Lb647.

Non-targeted metabolomic analysis revealed that different fermentation modes markedly reshaped the metabolite composition, abundance distribution, and dynamic succession patterns of hawthorn pulp. Among them, mixed fermentation exhibited the most pronounced advantages in expanding metabolic diversity and enriching key functional metabolites. As shown in the classification of metabolite categories in Figure 3A, the identified metabolites in hawthorn pulp were mainly grouped into organic acids and their derivatives, amino acids and related derivatives, phenolic compounds and their metabolites, lipids, sugars and sugar alcohols, as well as minor amounts of alkaloids and nucleosides. Organic acids and amino acid-related metabolites dominated the overall metabolic profile, reflecting that both the fruit matrix and lactic acid bacterial fermentation are primarily driven by carbon and nitrogen metabolism. Compared with the control group CK, the mixed fermentation samples showed an increase in the relative abundance of phenolic compounds and their derivatives, accompanied by a relative reduction in some primary metabolites, indicating a fermentation-induced shift from basic metabolic turnover toward the accumulation of functionally relevant secondary metabolites [34]. Combined with the targeted analysis, it revealed substantial changes in malic acid, citric acid, and lactic acid during fermentation. These observations were consistent with the metabolomics results, which showed significant enrichment of pathways related to pyruvate metabolism, the tricarboxylic acid cycle, and organic acid metabolism. The coordinated changes suggest that carbon flux redistribution during fermentation contributed to the dynamic transformation of organic acids observed in hawthorn pulp. Free amino acid accumulation observed during fermentation was further supported by the metabolomic data. Several differential metabolites were enriched in amino acid biosynthesis and amino acid metabolism pathways, indicating active nitrogen metabolism during microbial growth. These metabolic changes may contribute to both nutritional improvement and the formation of flavor precursor compounds. In addition, the increase in phenolic compounds was consistent with the enrichment of phenylpropanoid-related pathways detected by metabolomics.

Hierarchical clustering analysis at the sample level in Figure 3B further illustrated the impact of different fermentation modes on the global metabolomic profile. The CK samples were clearly separated from all fermented samples in the dendrogram, indicating that lactic acid bacterial fermentation was the primary driver of metabolic differentiation. Among the fermented samples, the mixed fermentation formed an independent cluster that was distinctly separated from both *S. thermophilus* 540 and *L. brevis* 647 monoculture fermentations. This pattern suggests that the metabolic outcome of co-fermentation was not a simple additive effect of the two single strains, but rather reflected the induction of a novel metabolic reprogramming mode arising from microbial interactions. The Venn diagram analysis in Figure 3C quantifies the differences in metabolite diversity among different fermentation modes. While a large number of shared metabolites were found among the CK, monoculture, and mixed fermentation groups, each group also possessed unique metabolites. Notably, metabolites specific to the control group were significantly reduced after mixed-culture fermentation; these metabolites may have been consumed and converted into substrates [35].

The PCA results in Figure 3D further confirmed the pronounced metabolic differences among the fermentation modes. The first two principal components together accounted for more than 60% of the total variance. PC1 mainly separated fermented samples from the unfermented control, while PC2 provided additional discrimination among the different fermentation strategies. Samples from the mixed fermentation clustered most tightly in the score plot and were clearly separated from both single strain fermentations, indicating higher stability and consistency of global metabolic regulation under co fermentation conditions. Clustering heatmaps of differential metabolites in Figure 3E–G provided deeper insight into these metabolic shifts. Compared with the control group (Mix vs. CK), mixed fermentation significantly upregulated a range of metabolites associated with antioxidant activity and flavor development, including chlorogenic acid derivatives, caffeic acid derivatives, and several aromatic amino acid related metabolites, with log2 fold changes generally exceeding 1. In parallel, several simple sugars and primary organic acids were downregulated, reflecting enhanced substrate conversion and metabolic utilization. When compared with *S. thermophilus* 540 (Mix vs. St540), mixed fermentation showed marked enrichment of phenolic metabolites and lipid related signaling molecules. In contrast, relative to *L. brevis* 647 (Mix vs. Lb647), mixed fermentation was characterized by a further accumulation of amino acid derived metabolites, highlighting the potentially complementary metabolic shifts of the two strains in shaping the overall metabolite profile of fermented hawthorn pulp [36]. Co-fermentation promotes the bio-metabolism of phenolic acid derivatives, amino acids, and lipid-related molecules, which may enhance the antioxidant capacity and flavor characteristics of the co-fermentation system.

### 3.8. Differential Metabolites and Their Dynamic Changes in Hawthorn Pulp Induced by Different Fermentation Modes

**Figure 4:**
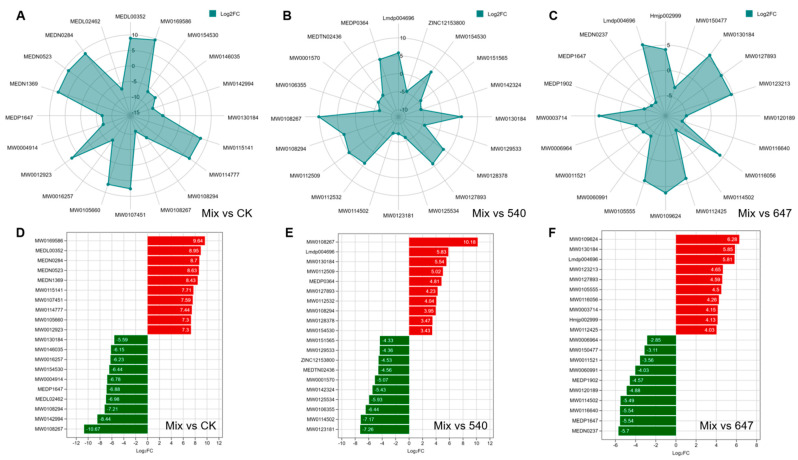

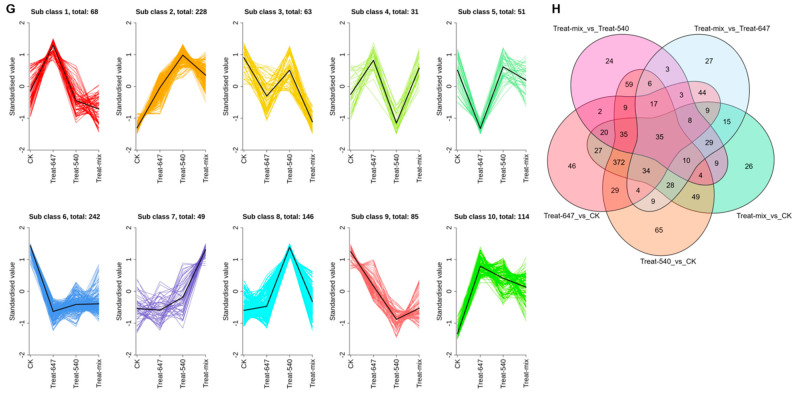
Differential metabolites and their dynamic changes in hawthorn pulp induced by different fermentation modes. Radar chart analysis of differential metabolites among groups with different fermentation methods (**A**) Mix vs. CK, (**B**) Mix vs. St540, (**C**) Mix vs. Lb647; Bar chart analysis of differential metabolites among groups with different fermentation methods (**D**) Mix vs. CK, (**E**) Mix vs. St540, (**F**) Mix vs. Lb647. (**G**) K-Means plot of differential metabolites. (**H**) Venn plot of differential groupings.

Radar plot analysis of differential metabolites (Figure 4A–C) showed that mixed fermentation exhibited significant differences across multiple metabolite categories compared with different reference groups. Compared with the unfermented control (Mix vs. CK), mixed fermentation displayed notable expansion in organic acids and their derivatives, phenolic compounds and their metabolites, as well as amino acids and their derivatives. Among these categories, phenolic related metabolites showed the largest variation amplitude, with relative contribution values markedly higher than those of other metabolite classes, indicating a pronounced enhancement of polyphenol related secondary metabolism during mixed fermentation. In the comparisons of Mix vs. St540 and Mix vs. Lb647, distinct patterns were observed. The Mix vs. St540 comparison was mainly characterized by further enrichment of phenolic and lipid metabolites, whereas the Mix vs. Lb647 comparison showed more pronounced differences in amino acid derivatives and organic acid related metabolites. These results indicate that mixed fermentation does not uniformly enhance all metabolic pathways, but instead exhibits differential metabolic modulation depending on the single strain fermentation system used as reference [37]. Co-fermentation promotes the bio-metabolism of phenolic acid derivatives, amino acids, and lipid-related molecules, which may enhance the antioxidant capacity and flavor characteristics of the co-fermentation system.

Bar chart analysis (Figure 4D–F) further quantified the number of differential metabolites and their upregulated and downregulated distributions among the different comparison groups. In Mix vs. CK, approximately 220 differential metabolites were identified, of which about 60 percent were upregulated, corresponding to roughly 130 metabolites, while around 90 metabolites were downregulated. In Mix vs. St540, the number of differential metabolites was approximately 150, with a relatively balanced distribution between upregulated and downregulated metabolites, although upregulated metabolites still showed a slight predominance at around 80 compounds. This result suggests that mixed fermentation further activates specific metabolic pathways beyond those induced by *S. thermophilus* fermentation alone. In Mix vs. Lb647, about 170 differential metabolites were detected, among which more than 100 were upregulated, clearly exceeding the number of downregulated metabolites. This indicates that, compared with *L. brevis* single fermentation, mixed fermentation leads to a more pronounced increase in metabolic complexity and overall metabolic activity [38].

K-means clustering analysis (Figure 4G) showed that differentially expressed metabolites could be roughly divided into three representative dynamic patterns: no significant difference, significant increase, and significant decrease. Differentially expressed metabolites were further divided into 10 trend subclasses. Subclasses 2, 7, 8, and 10 showed a continuous upward trend, with expression levels gradually increasing from the control group to the mixed treatment group, indicating that these metabolites may exhibit a time-dependent accumulation response. Subclasses 3, 6, and 9 showed a significant decrease in expression levels across all treatment groups, suggesting that these metabolites may be utilized as basal metabolites. The increased proportion of metabolites in the later stages of the mixed fermentation group may be due to the high metabolic activity maintained by microbial interactions in the later stages of the system, which is conducive to the continuous generation of functional compounds [39]. Venn diagram analysis of the differentially expressed metabolites (Figure 4H) showed that the mixed fermentation group had the most unique differentially expressed metabolites compared to single-strain fermentation and the control group, significantly exceeding the number of strain-specific metabolites observed in single fermentation. In contrast, the number of shared differentially expressed metabolites among the three groups was relatively limited.

## 4. Conclusions

This study comprehensively demonstrated that different lactic acid bacteria fermentation modes distinctly reshape the compositional and metabolic profiles of hawthorn pulp. Compared with the unfermented control, all fermentation strategies significantly enhanced biochemical transformation, whereas mixed fermentation (*Streptococcus thermophilus* 540 and *Lactobacillus brevis* 647, 1:1) induced the most pronounced metabolic changes. Mixed fermentation markedly increased total volatile compounds (231.14 μg/L vs. 49.80 μg/L in CK), particularly esters (41.45 μg/L vs. 9.88 μg/L), while maintaining a more balanced organic acid profile compared with single-strain fermentation. Untargeted metabolomics identified approximately 220 differential metabolites in Mix vs. CK, with nearly 60% upregulated. The mixed system exhibited the highest number of unique metabolites and a clear enrichment of phenolic derivatives, aromatic amino acid-related metabolites, and lipid-associated regulatory molecules, indicating activation of polyphenol-related secondary metabolism and intensified substrate conversion. Dynamic clustering analysis further revealed that mixed fermentation sustained higher metabolic activity during mid- and late-fermentation stages, which will be beneficial to the continuous accumulation of antioxidant-related metabolites and enhanced metabolic stability. Importantly, the observed changes were not a simple additive effect of single strains, but reflected metabolic complementarity and cross-substrate utilization between strains. Overall, this study provides mechanistic evidence that mixed lactic acid bacteria fermentation is an effective strategy to optimize bioactive enrichment, flavor complexity, and metabolic functionality in hawthorn pulp, providing preliminary insights into strain–matrix interactions during co-fermentation in this specific fruit system. Future studies integrating metagenomics, transcriptomics, or isotope tracing will be required to validate the microbial interaction mechanisms underlying these observed metabolic shifts.

## Figures and Tables

**Table 1:** The dynamic changes in physicochemical indicators during microbial fermentation.

Time (h)	Group	pH	SSC (Brix °)	Acids (g/L)	Viable Count (CFU/mL)	Reducing Sugar (mg/mL)	Total Phenol (mg/mL)	Total Flavonoid (mg/mL)
0	CK	3.96 ± 0.03 ^a^	13.32 ± 0.15 ^a^	5.19 ± 0.13 ^f^	0.00 ± 0.00 ^g^	13.59 ± 0.14 ^a^	3.08 ± 0.22 ^a^	2.89 ± 0.16 ^i^
6	St540	3.72 ± 0.20 ^b^	12.69 ± 0.10 ^bcd^	7.17 ± 0.07 ^e^	9.16 ± 0.19 ^cdef^	13.21 ± 0.18 ^b^	3.21 ± 0.13 ^a^	4.92 ± 0.21 ^defgh^
	Lb647	3.64 ± 0.04 ^b^	11.20 ± 0.06 ^f^	8.01 ± 0.18 ^d^	9.17 ± 0.35 ^cdef^	11.53 ± 0.11 ^c^	3.07 ± 0.09 ^a^	6.20 ± 0.12 ^a^
	1:1	3.72 ± 0.02 ^b^	12.85 ± 0.11 ^bc^	7.11 ± 0.20 ^e^	9.14 ± 0.07 ^cdef^	11.66 ± 0.10 ^c^	3.16 ± 0.11 ^a^	5.17 ± 0.10 ^cdef^
12	St540	3.68 ± 0.26 ^b^	12.62 ± 0.08 ^cde^	7.74 ± 0.07 ^d^	9.97 ± 0.29 ^a^	9.74 ± 0.10 ^fg^	3.05 ± 0.06 ^a^	5.20 ± 0.10 ^cde^
	Lb647	3.62 ± 0.08 ^b^	12.62 ± 0.07 ^cde^	8.79 ± 0.11 ^c^	9.30 ± 0.22 ^bcde^	10.77 ± 0.14 ^d^	3.24 ± 0.06 ^a^	5.17 ± 0.07 ^cdef^
	1:1	3.68 ± 0.03 ^b^	12.66 ± 0.16 ^bcde^	6.97 ± 0.19 ^e^	8.97 ± 0.22 ^def^	9.28 ± 0.21 ^h^	3.12 ± 0.23 ^a^	5.44 ± 0.11 ^bc^
18	St540	3.56 ± 0.09 ^b^	12.33 ± 0.05 ^e^	8.71 ± 0.09 ^c^	9.23 ± 0.31 ^bcdef^	9.43 ± 0.11 ^gh^	3.09 ± 0.22 ^a^	5.17 ± 0.06 ^cdef^
	Lb647	3.53 ± 0.04 ^b^	12.75 ± 0.04 ^bcd^	8.80 ± 0.09 ^c^	9.73 ± 0.32 ^abc^	10.43 ± 0.10 ^e^	3.16 ± 0.20 ^a^	4.83 ± 0.08 ^fgh^
	1:1	3.57 ± 0.04 ^b^	12.42 ± 0.13 ^de^	8.58 ± 0.24 ^c^	9.82 ± 0.25 ^ab^	8.60 ± 0.12 ^i^	3.16 ± 0.09 ^a^	4.97 ± 0.24 ^defg^
24	St540	3.54 ± 0.04 ^b^	12.67 ± 0.11 ^bcde^	8.75 ± 0.14 ^c^	8.79 ± 0.29 ^def^	9.32 ± 0.09 ^h^	3.11 ± 0.09 ^a^	5.14 ± 0.09 ^cdef^
	Lb647	3.50 ± 0.05 ^b^	12.82 ± 0.04 ^bc^	8.92 ± 0.20 ^c^	8.66 ± 0.26 ^f^	9.88 ± 0.13 ^f^	3.15 ± 0.10 ^a^	5.04 ± 0.13 ^defg^
	1:1	3.56 ± 0.05 ^b^	12.71 ± 0.17 ^bcd^	8.72 ± 0.20 ^c^	9.45 ± 0.27 ^abcd^	8.49 ± 0.16 ^i^	3.26 ± 0.16 ^a^	5.25 ± 0.11 ^cd^
30	St540	3.52 ± 0.14 ^b^	12.73 ± 0.07 ^bcd^	9.60 ± 0.18 ^b^	9.15 ± 0.32 ^cdef^	9.22 ± 0.08 ^h^	3.10 ± 0.13 ^a^	5.13 ± 0.13 ^cdef^
	Lb647	3.48 ± 0.03 ^b^	12.86 ± 0.05 ^bc^	9.56 ± 0.12 ^b^	8.64 ± 0.39 ^ef^	9.68 ± 0.13 ^fg^	3.29 ± 0.07 ^a^	4.78 ± 0.09 ^gh^
	1:1	3.48 ± 0.07 ^b^	12.75 ± 0.34 ^bcd^	9.44 ± 0.23 ^b^	9.10 ± 0.34 ^cdef^	8.54 ± 0.34 ^i^	3.25 ± 0.12 ^a^	4.89 ± 0.24 ^efgh^
36	St540	3.51 ± 0.14 ^b^	12.86 ± 0.05 ^bc^	9.45 ± 0.12 ^b^	8.82 ± 0.29 ^def^	9.16 ± 0.08 ^h^	3.22 ± 0.17 ^a^	5.72 ± 0.10 ^b^
	Lb647	3.49 ± 0.07 ^b^	12.91 ± 0.03 ^bc^	10.27 ± 0.06 ^a^	8.58 ± 0.40 ^f^	9.46 ± 0.09 ^gh^	3.11 ± 0.08 ^a^	4.96 ± 0.16 ^defg^
	1:1	3.52 ± 0.02 ^b^	13.00 ± 0.38 ^bc^	10.07 ± 0.19 ^a^	9.02 ± 0.14 ^def^	8.37 ± 0.21 ^i^	3.26 ± 0.08 ^a^	4.62 ± 0.24 ^h^

Results are expressed as mean ± standard deviation of three independent trials. Different lowercase letters in the same column represent significant differences (*p* < 0.05).

**Table 2:** The dynamic changes in organic acid content during microbial fermentation.

Time (h)	Group	Malic Acid (mg/L)	Citric Acid (mg/L)	Tartaric Acid (mg/L)	Lactic Acid (mg/L)	Oxalic Acid (mg/L)	Shikimic Acid (mg/L)	Quinic Acid (mg/L)
0	CK	514.13 ± 9.12 ^a^	356.59 ± 9.80 ^c^	814.70 ± 18.37 ^d^	0.00 ± 0.00 ^p^	29.53 ± 1.34 ^h^	29.38 ± 2.44 ^efg^	27.34 ± 0.56 ^cd^
6	St540	0.00 ± 0.00 ^m^	100.16 ± 4.92 ^fg^	921.15 ± 2.67 ^a^	341.72 ± 7.36 ^o^	75.80 ± 1.43 ^a^	5.12 ± 0.27 ^j^	31.73 ± 1.20 ^a^
	Lb647	42.34 ± 1.53 ^l^	97.55 ± 1.23 ^fgh^	582.12 ± 4.73 ^j^	515.35 ± 9.72 ^n^	75.55 ± 1.36 ^a^	9.50 ± 0.33 ^i^	18.62 ± 1.29 ^h^
	1:1	0.00 ± 0.00 ^m^	100.69 ± 3.17 ^f^	926.33 ± 3.88 ^a^	564.42 ± 2.30 ^m^	68.87 ± 1.40 ^b^	2.01 ± 0.12 ^j^	31.77 ± 1.35 ^a^
12	St540	209.15 ± 4.22 ^h^	89.79 ± 1.17 ^fghi^	751.52 ± 3.09 ^f^	927.92 ± 4.54 ^i^	45.30 ± 1.08 ^f^	9.80 ± 0.10 ^i^	25.04 ± 0.79 ^ef^
	Lb647	46.38 ± 0.75 ^l^	386.38 ± 10.93 ^b^	668.23 ± 3.67 ^i^	675.24 ± 6.73 ^l^	59.93 ± 1.81 ^c^	19.73 ± 1.29 ^h^	21.82 ± 0.93 ^g^
	1:1	207.83 ± 2.49 ^h^	95.51 ± 2.71 ^fgh^	868.48 ± 7.26 ^b^	764.20 ± 3.75 ^k^	24.76 ± 0.66 ^i^	11.43 ± 0.19 ^i^	29.89 ± 1.11 ^ab^
18	St540	273.35 ± 5.34 ^ef^	88.51 ± 1.18 ^ghi^	752.62 ± 7.39 ^f^	987.33 ± 5.86 ^h^	74.68 ± 2.40 ^a^	28.51 ± 0.99 ^fg^	25.29 ± 1.09 ^def^
	Lb647	85.05 ± 2.57 ^k^	456.21 ± 13.15 ^a^	673.67 ± 9.38 ^hi^	816.24 ± 13.67 ^j^	46.32 ± 1.30 ^f^	32.11 ± 0.77 ^e^	22.37 ± 0.91 ^g^
	1:1	277.81 ± 6.27 ^de^	98.05 ± 2.57 ^fgh^	837.04 ± 6.40 ^c^	952.12 ± 6.82 ^i^	54.85 ± 1.46 ^d^	26.32 ± 0.72 ^g^	28.41 ± 0.82 ^bc^
24	St540	355.18 ± 5.32 ^b^	87.39 ± 1.50 ^hi^	711.02 ± 15.96 ^g^	1164.24 ± 23.14 ^g^	49.41 ± 2.24 ^e^	37.43 ± 1.22 ^d^	23.40 ± 0.49 ^fg^
	Lb647	118.88 ± 5.49 ^j^	137.68 ± 1.66 ^d^	704.08 ± 5.72 ^g^	1273.56 ± 6.03 ^f^	35.16 ± 1.47 ^g^	36.75 ± 1.24 ^d^	23.51 ± 1.11 ^fg^
	1:1	260.95 ± 9.53 ^g^	90.69 ± 2.18 ^fghi^	811.14 ± 5.61 ^d^	1325.49 ± 19.50 ^e^	22.11 ± 0.90 ^i^	30.21 ± 1.03 ^ef^	27.36 ± 0.78 ^cd^
30	St540	263.80 ± 3.18 ^fg^	82.88 ± 1.62 ^i^	656.42 ± 7.79 ^i^	1575.94 ± 20.49 ^c^	28.97 ± 0.81 ^h^	46.83 ± 1.64 ^bc^	21.55 ± 0.62 ^g^
	Lb647	134.88 ± 3.71 ^i^	126.46 ± 3.42 ^e^	669.73 ± 6.64 ^i^	1457.08 ± 8.01 ^d^	37.59 ± 1.35 ^g^	47.76 ± 1.71 ^b^	22.34 ± 0.57 ^g^
	1:1	311.85 ± 7.90 ^c^	94.46 ± 1.25 ^fghi^	777.96 ± 5.94 ^e^	1468.24 ± 12.86 ^d^	28.32 ± 1.32 ^h^	44.36 ± 2.06 ^c^	26.45 ± 0.85 ^cde^
34	St540	287.35 ± 5.76 ^d^	90.67 ± 1.39 ^fghi^	687.08 ± 4.91 ^h^	1917.74 ± 27.57 ^a^	29.45 ± 1.22 ^h^	49.49 ± 2.15 ^b^	22.51 ± 1.21 ^g^
	Lb647	215.67 ± 2.69 ^h^	93.85 ± 1.81 ^fghi^	844.02 ± 7.25 ^c^	1799.43 ± 9.98 ^b^	51.68 ± 1.35 ^e^	56.28 ± 1.46 ^a^	28.49 ± 0.64 ^bc^
	1:1	317.48 ± 1.99 ^c^	95.36 ± 0.88 ^fgh^	801.46 ± 4.12 ^d^	1934.98 ± 12.38 ^a^	34.81 ± 1.48 ^g^	48.76 ± 3.85 ^b^	27.29 ± 1.12 ^cd^

Results are expressed as mean ± standard deviation of three independent trials. Different lowercase letters in the same column represent significant differences (*p* < 0.05).

**Table 3:** Dynamic changes of monomeric phenols during microbial fermentation.

Time (h)	Group	Chlorogenic Acid (mg/L)	Catechin (mg/L)	Caffeic Acid (mg/L)	Epicatechin (mg/L)	Rutin (mg/L)	Ferulic Acid (mg/L)	Kaempferol (mg/L)
0	CK	18.67 ± 0.22 ^def^	33.89 ± 0.69 ^a^	58.41 ± 0.97 ^bcd^	73.53 ± 0.75 ^bcd^	13.95 ± 0.75 ^b^	0.00 ± 0.00 ^f^	198.54 ± 2.02 ^d^
6	St540	47.67 ± 1.65 ^bc^	30.24 ± 0.61 ^b^	6.88 ± 0.71 ^ij^	65.12 ± 1.72 ^e^	15.40 ± 0.86 ^ab^	13.36 ± 0.91 ^abc^	148.33 ± 1.76 ^k^
	Lb647	47.14 ± 0.72 ^c^	30.66 ± 0.51 ^b^	11.73 ± 0.49 ^h^	75.41 ± 1.08 ^b^	15.45 ± 0.75 ^ab^	13.07 ± 0.65 ^abcd^	162.80 ± 2.21 ^j^
	1:1	51.37 ± 1.09 ^a^	25.36 ± 0.67 ^def^	10.46 ± 0.53 ^h^	193.23 ± 2.38 ^a^	15.52 ± 0.66 ^ab^	11.61 ± 0.65 ^de^	307.22 ± 2.32 ^a^
12	St540	19.78 ± 0.95 ^d^	26.49 ± 1.75 ^cd^	62.87 ± 1.87 ^a^	74.15 ± 0.88 ^bc^	14.83 ± 0.67 ^ab^	12.53 ± 0.64 ^bcde^	210.50 ± 2.10 ^c^
	Lb647	49.19 ± 0.70 ^b^	27.44 ± 0.80 ^c^	56.29 ± 1.04 ^defg^	57.63 ± 1.61 ^h^	14.29 ± 0.67 ^b^	12.19 ± 0.55 ^cde^	216.78 ± 2.51 ^b^
	1:1	47.31 ± 0.77 ^c^	24.42 ± 0.61 ^ef^	7.85 ± 0.61 ^i^	64.76 ± 1.55 ^ef^	15.36 ± 0.63 ^ab^	13.70 ± 0.54 ^abc^	148.51 ± 1.85 ^k^
18	St540	18.24 ± 0.60 ^defg^	26.11 ± 0.81 ^cde^	5.62 ± 0.49 ^j^	62.95 ± 0.86 ^ef^	14.45 ± 0.89 ^b^	12.35 ± 1.07 ^bcde^	139.85 ± 2.65 ^l^
	Lb647	18.41 ± 0.69 ^defg^	30.43 ± 1.02 ^b^	5.81 ± 0.61 ^j^	71.17 ± 1.39 ^cd^	14.55 ± 0.45 ^b^	11.02 ± 0.78 ^e^	152.38 ± 3.24 ^k^
	1:1	18.96 ± 0.66 ^de^	23.56 ± 0.89 ^f^	56.91 ± 0.87 ^cde^	61.78 ± 1.43 ^fg^	14.89 ± 0.59 ^ab^	13.00 ± 0.57 ^abcd^	189.29 ± 2.36 ^e^
24	St540	16.78 ± 0.99 ^fgh^	20.11 ± 0.83 ^g^	58.79 ± 0.97 ^bc^	70.81 ± 1.15 ^d^	16.47 ± 0.59 ^a^	14.28 ± 1.02 ^a^	177.51 ± 1.64 ^g^
	Lb647	15.38 ± 0.60 ^h^	25.12 ± 0.45 ^def^	57.81 ± 1.27 ^bcde^	65.60 ± 1.77 ^e^	15.05 ± 0.67 ^ab^	12.28 ± 0.51 ^bcde^	166.34 ± 1.89 ^j^
	1:1	18.48 ± 0.36 ^def^	15.45 ± 0.69 ^i^	55.70 ± 0.66 ^efg^	57.85 ± 1.28 ^h^	14.61 ± 0.47 ^b^	13.04 ± 0.67 ^abcd^	175.35 ± 0.98 ^gh^
30	St540	17.59 ± 1.09 ^efg^	17.82 ± 0.70 ^h^	55.61 ± 1.43 ^efg^	58.65 ± 1.41 ^h^	14.49 ± 0.90 ^b^	12.21 ± 0.35 ^cde^	176.53 ± 0.91 ^g^
	Lb647	17.58 ± 0.57 ^efg^	20.36 ± 0.89 ^g^	56.78 ± 0.98 ^cdef^	59.62 ± 1.93 ^gh^	14.68 ± 0.59 ^b^	13.94 ± 0.75 ^ab^	183.13 ± 2.38 ^f^
	1:1	17.07 ± 0.74 ^efgh^	16.26 ± 0.48 ^hi^	59.17 ± 0.94 ^b^	65.59 ± 0.86 ^e^	14.92 ± 0.66 ^ab^	14.39 ± 0.89 ^a^	171.31 ± 2.56 ^hi^
34	St540	16.42 ± 1.12 ^gh^	10.32 ± 0.85 ^k^	57.38 ± 0.95 ^bcde^	64.92 ± 1.30 ^e^	15.16 ± 0.95 ^ab^	13.19 ± 0.71 ^abcd^	167.10 ± 1.56 ^ij^
	Lb647	17.31 ± 0.57 ^efg^	12.14 ± 0.86 ^j^	54.15 ± 0.72 ^g^	52.17 ± 1.68 ^i^	14.60 ± 0.98 ^b^	13.07 ± 0.85 ^abcd^	163.40 ± 2.60 ^j^
	1:1	17.38 ± 1.09 ^efg^	16.72 ± 0.61 ^hi^	54.71 ± 0.92 ^fg^	58.27 ± 0.73 ^h^	14.95 ± 0.72 ^ab^	13.22 ± 0.57 ^abcd^	176.43 ± 1.94 ^g^

Results are expressed as mean ± standard deviation of three independent trials. Different lowercase letters in the same column represent significant differences (*p* < 0.05).

**Table 4:** Dynamic changes of free amino acids during microbial fermentation.

Time (h)	Group	L-Asp (mg/L)	L-Thr (mg/L)	L-Ser (mg/L)	L-Glu (mg/L)	L-Pro (mg/L)	Gly (mg/L)	L-Ala (mg/L)	L-Val (mg/L)	L-Met (mg/L)	L-Ile (mg/L)	L-Leu (mg/L)	L-Tyr (mg/L)	L-Phe (mg/L)	L-His (mg/L)	L-Lys (mg/L)	L-Arg (mg/L)
0	CK	2.656 ± 0.133 ^a^	1.111 ± 0.083 ^a^	1.093 ± 0.094 ^a^	3.321 ± 0.174 ^a^	1.083 ± 0.094 ^a^	1.130 ± 0.104 ^a^	1.244 ± 0.131 ^b^	1.316 ± 0.179 ^a^	0.265 ± 0.028 ^a^	1.103 ± 0.082 ^a^	1.694 ± 0.121 ^a^	0.735 ± 0.108 ^a^	1.117 ± 0.095 ^a^	0.570 ± 0.100 ^a^	1.706 ± 0.069 ^bc^	1.080 ± 0.099 ^a^
6	St540	2.691 ± 0.087 ^a^	1.104 ± 0.058 ^a^	1.104 ± 0.039 ^a^	3.365 ± 0.155 ^a^	1.107 ± 0.055 ^a^	1.122 ± 0.050 ^a^	1.263 ± 0.073 ^b^	1.297 ± 0.070 ^a^	0.267 ± 0.046 ^a^	1.116 ± 0.056 ^a^	1.685 ± 0.065 ^a^	0.745 ± 0.044 ^a^	1.104 ± 0.065 ^a^	0.589 ± 0.061 ^a^	1.895 ± 0.070 ^a^	1.085 ± 0.049 ^a^
	Lb647	2.696 ± 0.112 ^a^	1.102 ± 0.040 ^a^	1.104 ± 0.058 ^a^	3.364 ± 0.134 ^a^	1.116 ± 0.038 ^a^	1.126 ± 0.016 ^a^	1.260 ± 0.047 ^b^	1.296 ± 0.072 ^a^	0.265 ± 0.049 ^a^	1.116 ± 0.023 ^a^	1.685 ± 0.073 ^a^	0.743 ± 0.072 ^a^	1.108 ± 0.013 ^a^	0.588 ± 0.061 ^a^	1.691 ± 0.078 ^c^	1.082 ± 0.087 ^a^
	1:1	2.693 ± 0.138 ^a^	1.098 ± 0.168 ^a^	1.108 ± 0.087 ^a^	3.364 ± 0.160 ^a^	1.113 ± 0.090 ^a^	1.122 ± 0.078 ^a^	1.263 ± 0.150 ^b^	1.297 ± 0.168 ^a^	0.274 ± 0.019 ^a^	1.120 ± 0.148 ^a^	1.683 ± 0.134 ^a^	0.743 ± 0.058 ^a^	1.111 ± 0.069 ^a^	0.588 ± 0.070 ^a^	1.691 ± 0.106 ^c^	1.083 ± 0.090 ^a^
12	St540	2.690 ± 0.162 ^a^	1.094 ± 0.078 ^a^	1.100 ± 0.007 ^a^	3.358 ± 0.123 ^a^	1.096 ± 0.080 ^a^	1.113 ± 0.048 ^a^	1.513 ± 0.091 ^a^	1.284 ± 0.065 ^a^	0.261 ± 0.049 ^a^	1.108 ± 0.095 ^a^	1.671 ± 0.094 ^a^	0.739 ± 0.046 ^a^	1.096 ± 0.082 ^a^	0.582 ± 0.058 ^a^	1.880 ± 0.069 ^ab^	1.082 ± 0.065 ^a^
	Lb647	2.685 ± 0.089 ^a^	1.094 ± 0.048 ^a^	1.091 ± 0.087 ^a^	3.353 ± 0.080 ^a^	1.104 ± 0.058 ^a^	1.115 ± 0.064 ^a^	1.510 ± 0.073 ^a^	1.285 ± 0.073 ^a^	0.260 ± 0.029 ^a^	1.118 ± 0.092 ^a^	1.681 ± 0.073 ^a^	0.734 ± 0.055 ^a^	1.095 ± 0.081 ^a^	0.575 ± 0.049 ^a^	1.881 ± 0.069 ^ab^	1.075 ± 0.083 ^a^
	1:1	2.684 ± 0.090 ^a^	1.092 ± 0.077 ^a^	1.100 ± 0.088 ^a^	3.357 ± 0.086 ^a^	1.109 ± 0.098 ^a^	1.119 ± 0.093 ^a^	1.156 ± 0.107 ^b^	1.287 ± 0.071 ^a^	0.260 ± 0.051 ^a^	1.111 ± 0.068 ^a^	1.671 ± 0.090 ^a^	0.740 ± 0.042 ^a^	1.107 ± 0.070 ^a^	0.582 ± 0.066 ^a^	1.881 ± 0.078 ^ab^	1.073 ± 0.079 ^a^
18	St540	2.677 ± 0.140 ^a^	1.084 ± 0.060 ^a^	1.087 ± 0.065 ^a^	3.339 ± 0.152 ^a^	1.085 ± 0.063 ^a^	1.084 ± 0.042 ^a^	1.497 ± 0.093 ^a^	1.273 ± 0.097 ^a^	0.246 ± 0.043 ^a^	1.091 ± 0.046 ^a^	1.664 ± 0.065 ^a^	0.726 ± 0.051 ^a^	1.084 ± 0.067 ^a^	0.568 ± 0.053 ^a^	1.875 ± 0.087 ^ab^	1.062 ± 0.060 ^a^
	Lb647	2.675 ± 0.098 ^a^	1.084 ± 0.074 ^a^	1.082 ± 0.095 ^a^	3.334 ± 0.143 ^a^	1.088 ± 0.085 ^a^	1.081 ± 0.086 ^a^	1.498 ± 0.090 ^a^	1.273 ± 0.087 ^a^	0.244 ± 0.038 ^a^	1.092 ± 0.087 ^a^	1.669 ± 0.108 ^a^	0.724 ± 0.047 ^a^	1.083 ± 0.073 ^a^	0.576 ± 0.071 ^a^	1.873 ± 0.066 ^ab^	1.065 ± 0.065 ^a^
	1:1	2.674 ± 0.092 ^a^	1.085 ± 0.091 ^a^	1.082 ± 0.071 ^a^	3.347 ± 0.085 ^a^	1.088 ± 0.078 ^a^	1.086 ± 0.076 ^a^	1.499 ± 0.095 ^a^	1.273 ± 0.089 ^a^	0.247 ± 0.035 ^a^	1.095 ± 0.081 ^a^	1.670 ± 0.111 ^a^	0.733 ± 0.034 ^a^	1.089 ± 0.076 ^a^	0.570 ± 0.021 ^a^	1.872 ± 0.071 ^ab^	1.069 ± 0.037 ^a^
24	St540	2.264 ± 0.091 ^b^	1.064 ± 0.049 ^a^	1.064 ± 0.066 ^a^	3.324 ± 0.071 ^a^	1.060 ± 0.053 ^a^	1.077 ± 0.047 ^a^	1.482 ± 0.078 ^a^	1.264 ± 0.091 ^a^	0.230 ± 0.038 ^a^	1.079 ± 0.059 ^a^	1.649 ± 0.042 ^a^	0.707 ± 0.062 ^a^	1.065 ± 0.058 ^a^	0.545 ± 0.042 ^a^	1.855 ± 0.066 ^abc^	1.046 ± 0.043 ^a^
	Lb647	2.652 ± 0.078 ^a^	1.065 ± 0.064 ^a^	1.094 ± 0.080 ^a^	3.323 ± 0.103 ^a^	1.063 ± 0.033 ^a^	1.073 ± 0.082 ^a^	1.483 ± 0.076 ^a^	1.263 ± 0.069 ^a^	0.228 ± 0.039 ^a^	1.079 ± 0.077 ^a^	1.652 ± 0.065 ^a^	0.704 ± 0.074 ^a^	1.063 ± 0.105 ^a^	0.545 ± 0.051 ^a^	1.857 ± 0.061 ^abc^	1.043 ± 0.047 ^a^
	1:1	2.655 ± 0.068 ^a^	1.064 ± 0.066 ^a^	1.072 ± 0.065 ^a^	3.329 ± 0.098 ^a^	1.063 ± 0.074 ^a^	1.076 ± 0.077 ^a^	1.486 ± 0.087 ^a^	1.260 ± 0.085 ^a^	0.231 ± 0.030 ^a^	1.080 ± 0.077 ^a^	1.649 ± 0.085 ^a^	0.710 ± 0.080 ^a^	1.077 ± 0.045 ^a^	0.549 ± 0.040 ^a^	1.855 ± 0.066 ^abc^	1.052 ± 0.056 ^a^
30	St540	2.666 ± 0.092 ^a^	1.075 ± 0.057 ^a^	1.075 ± 0.065 ^a^	3.341 ± 0.090 ^a^	1.091 ± 0.068 ^a^	1.098 ± 0.080 ^a^	1.525 ± 0.087 ^a^	1.285 ± 0.091 ^a^	0.241 ± 0.046 ^a^	1.219 ± 0.052 ^a^	1.749 ± 0.055 ^a^	0.799 ± 0.069 ^a^	1.101 ± 0.063 ^a^	0.586 ± 0.051 ^a^	1.878 ± 0.085 ^ab^	1.071 ± 0.071 ^a^
	Lb647	2.664 ± 0.107 ^a^	1.073 ± 0.099 ^a^	1.078 ± 0.085 ^a^	3.336 ± 0.167 ^a^	1.083 ± 0.024 ^a^	1.094 ± 0.081 ^a^	1.521 ± 0.072 ^a^	1.278 ± 0.072 ^a^	0.244 ± 0.040 ^a^	1.201 ± 0.071 ^a^	1.746 ± 0.075 ^a^	0.821 ± 0.067 ^a^	1.090 ± 0.086 ^a^	0.577 ± 0.061 ^a^	1.877 ± 0.060 ^ab^	1.068 ± 0.076 ^a^
	1:1	2.662 ± 0.075 ^a^	1.071 ± 0.074 ^a^	1.076 ± 0.079 ^a^	3.334 ± 0.093 ^a^	1.075 ± 0.047 ^a^	1.083 ± 0.079 ^a^	1.505 ± 0.071 ^a^	1.275 ± 0.074 ^a^	0.241 ± 0.044 ^a^	1.195 ± 0.091 ^a^	1.743 ± 0.091 ^a^	0.818 ± 0.037 ^a^	1.082 ± 0.052 ^a^	0.563 ± 0.049 ^a^	1.865 ± 0.060 ^ab^	1.065 ± 0.112 ^a^

Results are expressed as mean ± standard deviation of three independent trials. Different lowercase letters in the same column represent significant differences (*p* < 0.05).

## Data Availability

The original contributions presented in this study are included in the article. Further inquiries can be directed to the corresponding authors.
